# Retraction and replacement: Stripes and loss of color in ball pythons (*Python regius*) are associated with variants affecting endothelin signaling

**DOI:** 10.1093/g3journal/jkad211

**Published:** 2023-09-30

This is a retraction and replacement of: Uyen M Dao, Izabella Lederer, Ray L Tabor, Basmah Shahid, Chiron W Graves, Hannah S Seidel, the BIO306W Consortium, Stripes and loss of color in ball pythons (*Python regius*) are associated with variants affecting endothelin signaling, *G3 Genes|Genomes|Genetics*, Volume 13, Issue 7, July 2023, jkad063, https://doi.org/10.1093/g3journal/jkad063

Shortly after article publication, the Authors of this article alerted the Editorial Office to the use of erroneous gene names for endothelin receptor genes. Details can be found in the Expression of Concern at https://doi.org/10.1093/g3journal/jkad182.

Given the nature of the changes and that no other results or conclusions were impacted by this error, the authors are retracting the original version of the article and replacing it with a new version, which has been accepted by the editors. The new version has been republished at the same location as the original article https://doi.org/10.1093/g3journal/jkad063.

